# Aggravated endothelial endocrine dysfunction and intimal thickening of renal artery in high-fat diet-induced obese pigs following renal denervation

**DOI:** 10.1186/s12872-020-01472-7

**Published:** 2020-04-16

**Authors:** Enyong Su, Linwei Zhao, Xiaohang Yang, Binbin Zhu, Yahui Liu, Wen Zhao, Xianpei Wang, Datun Qi, Lijie Zhu, Chuanyu Gao

**Affiliations:** 1 Department of Cardiology, Zhengzhou University People’s Hospital, No.7 Weiwu road, Jinshui District, Zhengzhou, 450003 Henan China; 2 Department of Cardiology, Huazhong Fuwai Hospital, Zhengzhou, 451464 Henan China; 3 Zhengzhou University School of Pharmaceutical Sciences, Zhengzhou, 450001 Henan China

**Keywords:** High-fat diet, Endothelial dysfunction, Renal denervation, Intimal thickening, Atherosclerosis, Hypertension

## Abstract

**Background:**

Renal denervation (RDN) targeting the sympathetic nerves in the renal arterial adventitia as a treatment of resistant hypertension can cause endothelial injury and vascular wall injury. This study aims to evaluate the risk of atherosclerosis induced by RDN in renal arteries.

**Methods:**

A total of 15 minipigs were randomly assigned to 3 groups: (1) control group, (2) sham group, and (3) RDN group (*n* = 5 per group). All pigs were fed a high-fat diet (HFD) for 6 months after appropriate treatment. The degree of intimal thickening of renal artery and the conversion of endothelin 1 (ET-1) receptors were evaluated by histological staining. Western blot was used to assess the expression of nitric oxide (NO) synthesis signaling pathway, ET-1 and its receptors, NADPH oxidase 2 (NOX2) and 4-hydroxynonenal (4-HNE) proteins, and the activation of NF-kappa B (NF-κB).

**Results:**

The histological staining results suggested that compared to the sham treatment, RDN led to significant intimal thickening and significantly promoted the production of endothelin B receptor (ET_B_R) in vascular smooth muscle cells (VSMCs). Western blotting analysis indicated that RDN significantly suppressed the expression of AMPK/Akt/eNOS signaling pathway proteins, and decreased the production of NO, and increased the expression of endothelin system proteins including endothelin-1 (ET-1), endothelin converting enzyme 1 (ECE1), endothelin A receptor (ET_A_R) and ET_B_R; and upregulated the expression of NOX2 and 4-HNE proteins and enhanced the activation of NF-kappa B (NF-κB) when compared with the sham treatment (all *p* < 0.05). There were no significant differences between the control and sham groups (all *p* > 0.05).

**Conclusions:**

RDN aggravated endothelial endocrine dysfunction and intimal thickening, and increased the risk of atherosclerosis in renal arteries of HFD-fed pigs.

## Background

Resistant hypertension, exhibiting elevated office blood pressure (BP, ≥130/80 mmHg), is a kind of common and complicated disease in clinical practice and is difficult to achieve control in patients despite treated with 3 or more different anti-hypertensive agents at best doses together including a diuretic according to the American Heart Association hypertension guidelines, which leads to increasing incidence of many complications such as renal injury, stroke and heart failure [1–3]. Research shows that sympathetic nerve overactivity is relevant to the development of hypertension [4]. Therefore, new therapeutics targeting sympathetic nerves are essential to treatment for resistant hypertension. Renal denervation (RDN) is an invasive technique for resistant hypertension via catheter-based radiofrequency ablation and has been shown to be effective in reducing BP in the Symplicity HTN-1 and Symplicity HTN-2 clinical trials [4, 5]. In addition, some studies have been conducted in clinical and preclinical animal models to assess the safety of the procedure, yet some problems, such as a short observation period, limited research scope including renal function, imaging and morphology of renal artery, exist [4–6]. The effects of the RDN procedure on the renal artery still need further study because radiofrequency energy is delivered transmurally and can cause vascular wall injury, which may cause endothelial dysfunction and result in an imbalanced release of an increased level of the endothelium-derived relaxation factor nitric oxide (NO) and a decreased level of the endothelium-derived constriction factor endothelin-1 (ET-1), thereby increasing the risk of atherosclerosis [7]. In addition, studies suggest a number of mechanisms of atherosclerosis, including the thrombosis theory, lipid infiltration theory, damage reaction hypothesis, oxidative stress hypothesis, immune dysfunction hypothesis, homocysteine hypothesis and inflammatory reaction theory [8, 9]. The vascular injury method combined with the use of a high-fat diet (HFD) may accelerate atherosclerosis progression [10, 11].

Based on these considerations, we studied the endothelial endocrine function of and intimal changes to the renal artery and aimed to evaluate the risk of adverse vascular outcomes after RDN in minipigs fed a HFD for 6 months.

## Methods

### Animals

All animal experimental protocols mentioned in this study were in accord with the Guidelines for the Care and Use of Laboratory Animals published by the US National Institutes of Health (NIH Publication No. 85–23, revised 1996) and were approved by the Ethics Committee of Zhengzhou University. Fifteen 8-month-old male Bama minipigs weighing 19–20 kg were provided by the Beijing Shi Chuang Century Minipig Breeding Base and were randomly divided into 3 groups: the control group (*n* = 5), the sham group (*n* = 5), and the RDN group (*n* = 5). All pigs were housed individually in pens with a suitable temperature (23 ± 1 °C) and humidity (50 ± 5%) and given access to a HFD (4100 kcal/kg) containing 10% protein, 41% fat, 43% carbohydrates and 6% minerals according to feeding guidelines at 5% of their body weight for 6 months after appropriate treatment and were then euthanized in deep anesthesia using propofol and bensulfatracurium by intravenous injection.

### Bilateral RDN procedure

Under anesthesia, the femoral arteries were punctured by the vascular incision method. A 7F sheath (Cordis Corporation, Florida, USA) was introduced into the artery and secured. Each pig was heparinized at 100 U/kg. The angiographic catheter (Cordis Corporation, Florida, USA) was inserted into the abdominal aortic region of the origin of the renal artery. In the RDN group, we performed renal arterial bilateral angiography to ascertain the renal artery location and to assess the feasibility of the RDN procedure. The angiographic catheter was withdrawn followed by insertion of the temperature-controlled cardiac radiofrequency catheter (NS7TCDL174HS, Biosense Webster, California, USA), which was connected to a generator (Johnson & Johnson, New Jersey, USA). Five radiofrequency ablation sites at intervals of 5 mm were selected, and RDN was performed bilaterally in a longitudinal and rotational manner from the distal to the proximal segments of the renal artery. The generator parameters were set as follows: energy, 8 W; and time at each site, 120 s [4]. The sham group underwent the same procedure except for the ablation, while the control group received no treatment.

After 6 months, all pigs were euthanized in deep anesthesia, and the ablated renal arteries from the RDN group and unablated renal arteries from the control and sham groups were obtained and processed for further analysis.

### Detection of metabolic profiles

Under anesthesia, the blood was collected from the superior vena cava of pigs using evacuated tubes, and then was centrifuged at 3000 r for 10 min in a desktop high-speed refrigerated centrifuge (Neofuge 15R, Heal Force, Shanghai, China) to separate the serum. Total cholesterol (TC), triglycerides (TG), high density lipoprotein cholesterol (HDL-C), low density lipoprotein cholesterol (LDL-C) and serum creatine (Scr) were measured by automatic biochemical analyzer (Chemray 240, Rayto, Shenzhen, China).

### Measurement of BP

To evaluate the effectiveness of RDN, systolic blood pressure (SBP) and diastolic blood pressure (DBP) of all pigs were measured by intelligent non-invasive sphygmomanometer (BP-2010E, Softron, Beijing, China) at baseline and at 2 days, 3 months and 6 months after different treatment. BP was taken 3 times and average figure would be available.

### Determination of NO and cGMP levels

After the renal arteries were homogenized, a colorimetric assay kit (No. A012–1, Jiancheng, Nanjing, China) was used according to the manufacturer’s protocol to detect the nitrite content in the supernatant, which was converted from nitrate by nitrite reductase, to reflect NO production. Cyclic guanosine monophosphate (cGMP) content in renal artery extracts was measured using a commercial immunoassay (No. E-EL-0083c, Elabscience, Wuhan, China).

### Immunoblotting

The frozen arteries were thoroughly homogenized, and total protein, cytoplasmic protein and nuclear protein were extracted. Subsequently, a BCA Protein Assay Kit (G2026, Servicebio, Wuhan, China) was used to detect protein concentration. Protein samples were separated by SDS-PAGE (10% gel), transferred to PDVF membranes and blocked with 5% skim milk dissolved in 0.5% TBST for 1 h. Then, the membranes were incubated with the primary antibodies rabbit polyclonal anti-NADPH oxidase 2 (NOX2; 1:1000; bs-3889R, Bioss, Beijing, China), rabbit polyclonal anti-4-hydroxynonenal (4-HNE; 1:1000; ab46545, Abcam, Cambridge, UK), mouse monoclonal anti-endothelin 1 (ET-1; 1:500; abx100923, Abbexa, Cambridge, UK), rabbit polyclonal anti-endothelin A receptor (ET_A_R; 1:1000; ab117521, Abcam, Cambridge, UK), rabbit polyclonal anti-endothelin B receptor (ET_B_R; 1:1000; ab117529, Abcam, Cambridge, UK), rabbit polyclonal anti-endothelin converting enzyme 1 (ECE1; 1:1000; bs-1190R, Bioss Beijing, China), rabbit polyclonal anti-adenosine 5′-monophosphate (AMP)-activated protein kinase alpha 1/2 (AMPK; 1:1000; abx008836, Abbexa, Cambridge, UK), rabbit polyclonal anti-phosphorylated AMPK alpha (Thr172) (1:1000; 2531, CST, Boston, USA), rabbit polyclonal anti-endothelial nitric oxide synthase (eNOS; 1:1000; ab5589, Abcam, Cambridge, UK), rabbit polyclonal anti-phosphorylated eNOS (Ser1177) (1:1000; 9571, CST, Boston, USA), rabbit polyclonal anti-protein kinase B (Akt; 1:1000; 9272, CST, Boston, USA), rabbit polyclonal anti-phosphorylated Akt (Ser473) (1:1000; 9271, CST, Boston, USA), mouse monoclonal anti-glyceraldehyde phosphate dehydrogenase (GAPDH; 1:25000; GB13002-m-1, Servicebio, Wuhan, China), mouse monoclonal anti-histone H3 (11,000; GB13102–1, Servicebio, Wuhan, China), rabbit monoclonal anti-phosphorylated-I kappa B alpha (Ser32) (p-IκB alpha;1:1000;2859, CST, Boston, USA) and rabbit polyclonal anti- phosphorylated NF-kappa B p65 (Ser529) (p-NF-κB p65; 1:1000; LS-B652–50, LSBio, Seattle, USA) overnight at 4 °C. After washing 3 times with TBST buffer, the membranes were incubated with horseradish peroxidase (HRP)-labeled goat anti-rabbit IgG (H + L) and HRP-labeled goat anti-mouse IgG (H + L) secondary antibodies for half an hour at room temperature. Immunoblotting was quantified by AlphaEaseFC software (Alpha Innotech, California, USA).

### Histopathology and immunohistochemistry

Renal arteries were fixed in 4% paraformaldehyde, washed, dehydrated by soaking in a graded ethanol series (75, 85, 90, 95 and 100%) and cleared in xylene. Vessels were paraffin-embedded and sliced into 5-μm sections at a 200-μm interval from the distal (kidney) to proximal (abdominal aorta) region for hematoxylin and eosin (HE) staining. For immunohistochemistry, sections were blocked in 3% bovine serum albumin (BSA) for 30 min and then incubated overnight at 4 °C with the primary antibody anti-ET_A_R (1:500; ab117521, Abcam, Cambridge, UK) or anti-ET_B_R (1:500; ab117529, Abcam, Cambridge, UK). Subsequently, the sections were washed, incubated with HRP-labeled goat anti-rabbit secondary antibody for 50 min at room temperature. The immunoreactions were developed with a diaminobenzidine (DAB) chromogenic kit. The nuclei were counterstained with HE.

### Statistical analysis

All data were evaluated with SPSS version 20.00 (International Business Machines Corporation, New York, USA) software. BP was indicated by mean ± standard error and other data were expressed as the mean ± standard deviation. Quantitative indicators were compared using the paired samples t-tests within group. Comparisons between groups were carried out using one-way analysis of variance (ANOVA), followed by the least significant difference (LSD) test to determine the statistical significance of the differences between means. *P* values less than 0.05 were regarded as statistically significant.

## Results

### Body weight and serum creatinine levels and lipid profiles at baseline and after 6 months

Compared with the baseline, the significant increases of body weight and TC and significant decrease of HDL-C were observed in pigs of control group (*p* = 0.000, *p* = 0.013, and *p* = 0.006, respectively), sham group (*p* = 0.000, *p* = 0.029, and *p* = 0.001, respectively) and RDN group (p = 0.000, p = 0.029, and *p* = 0.025, respectively) after a 6-month HFD. There was no statistical difference of body weight, Scr, TC, TG, HDL-C and LDL-C between RND group and sham group after 6 months (*p* = 0.194, *p* = 0.418, *p* = 0.890, *p* = 0.686, *p* = 0.728 and *p* = 0.287, respectively, Table 1).

**Table 1:** Levels of body weight and Scr and lipid profiles

	Baseline			After 6 months		
	Control group	Sham group	RDN group	Control group	Sham group	RDN group
TC (mmol/L)	2.71 ± 0.17	2.75 ± 0.16	2.80 ± 0.14	3.20 ± 0.16^#^	3.34 ± 0.23^#^	3.23 ± 0.18^#^
TG (mmol/L)	1.40 ± 0.14	1.37 ± 0.17	1.35 ± 0.21	1.35 ± 0.17	1.44 ± 0.12	1.40 ± 0.17
HDL-C (mmol/L)	1.41 ± 0.13	1.41 ± 0.11	1.38 ± 0.13	1.15 ± 0.10^##^	1.08 ± 0.04^##^	1.11 ± 0.06^#^
LDL-C (mmol/L)	2.63 ± 0.17	2.56 ± 0.12	2.62 ± 0.13	2.63 ± 0.09	2.54 ± 014	2.63 ± 0.12
Scr (umol/L)	80.57 ± 6.98	78.29 ± 7.10	77.60 ± 3.27	83.78 ± 5.85	80.07 ± 5.78	82.98 ± 4.72
Body weight (kg)	24.80 ± 2.97	24.40 ± 2.21	25.20 ± 1.75	68.3 ± 1.44^###^	68.00 ± 1.54^###^	66.60 ± 1.82^###^

Data are expressed as the mean ± standard deviation. ^#^p < 0.05, ^##^p < 0.01, ^###^p < 0.001 vs. baseline; n = 5 per group. Abbreviations: RDN, renal denervation; TC, total cholesterol; TG, triglyceride; HDL-C, high density lipoprotein cholesterol; LDL-C, low density lipoprotein cholesterol; Scr, serum creatinine

### Changes of BP in pigs with and without RDN

**Fig. 1:**
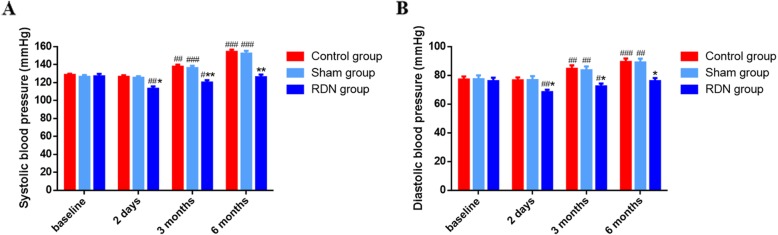
Effects of RDN treatment on BP at different time. Changes of (**a**) SBP and (**b**) DBP in pigs treated differently after 2 days, 3 months and 6 months. Data are expressed as the mean ± standard error. ^#^*p* < 0.05, ^##^*p* < 0.01, ^###^*p* < 0.001 vs. baseline; *p < 0.05, **p < 0.01 vs. sham group; *n* = 5 per group. Abbreviations: RDN, renal denervation; BP, blood pressure; SBP, systolic blood pressure; DBP, diastolic blood pressure

After a HFD for 3 and 6 months, SBP and DBP were significantly elevated in pigs of control group (*p* = 0.005, p = 0.000, *p* = 0.002 and p = 0.000, respectively) and sham group (p = 0.000, p = 0.000, *p* = 0.008 and *p* = 0.003, respectively) when compared with baseline BP. SBP and DBP significantly decreased post-RDN for 2 days in RDN group (p = 0.002 and *p* = 0.001, respectively), while no significant difference was found in control group (*p* = 0.051 and *p* = 0.553, respectively) and sham group (*p* = 0.230 and *p* = 0.553, respectively) which underwent different treatment for 2 days. In comparison with sham group, RDN group had apparently lower SBP (*p* = 0.010, *p* = 0.004 and *p* = 0.006, respectively) and DBP (*p* = 0.039, *p* = 0.038, and *p* = 0.031, respectively) after a HFD for 2 days, 3 months and 6 months (Fig. 1).

### Pathologic examinations

**Fig. 2:**
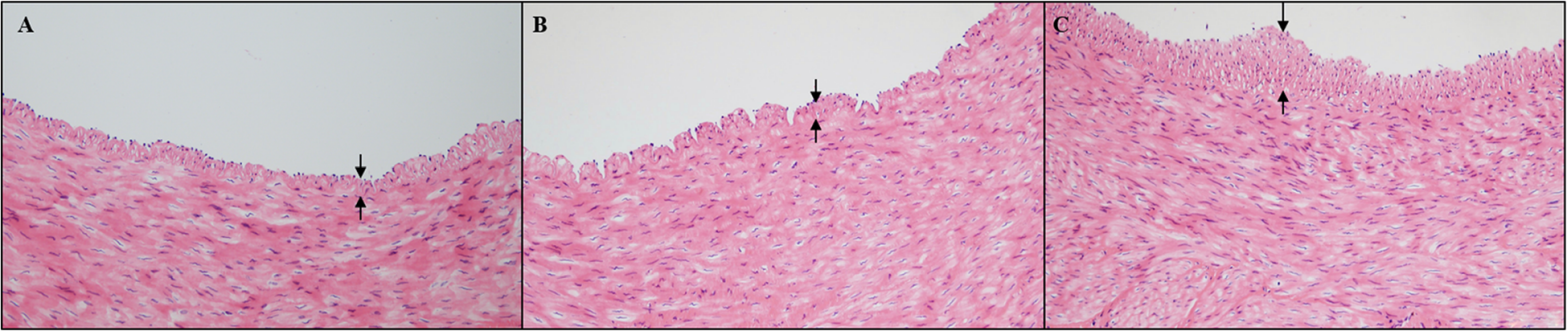
Representative images of intima stained by HE in the 3 groups (× 200). **a** Control group, (**b**) sham group, (**c**) RDN group. Black double arrows indicate thickening of the intima. Abbreviations: HE, hematoxylin and eosin; RDN, renal denervation

HE staining showed intimal thickening and irregular arrangement of vascular smooth muscle cells (VSMCs) in all 3 groups after a HFD for 6 months. The degree of intimal thickening in the control group and the sham group was comparable (Fig. 2a and b). In comparison with the sham group, the RDN group had significantly increased intimal thickness (Fig. 2b and c).

### Protein expression of the NADPH oxidase subunit NOX2 and 4-HNE in ablated and unablated renal arteries

**Fig. 3:**
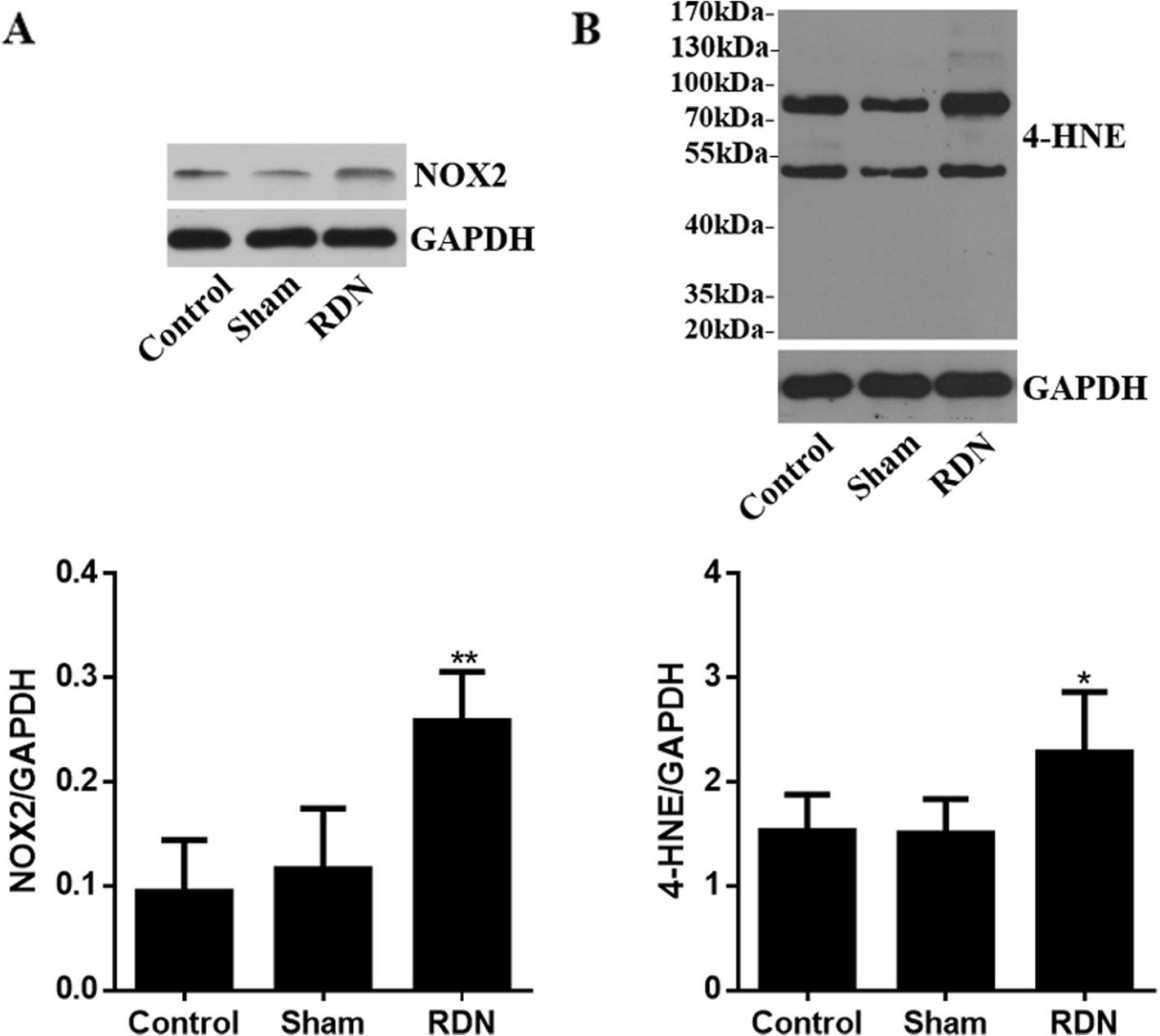
Effects of RDN treatment on NOX2 and 4-HNE expression. **a** NOX2, (**b**) 4-HNE. Data are expressed as the mean ± standard deviation. *p < 0.05, **p < 0.01 vs. sham group; *n* = 5 per group. Abbreviations: RDN, renal denervation; NOX2, NADPH oxidase 2; 4-HNE,4-hydroxynonenal; GAPDH, glyceraldehyde phosphate dehydrogenase

Compared to the sham group, the RDN group had significantly increased protein expression of the NADPH oxidase catalytic subunit NOX2 and of 4-HNE resulting from lipid peroxidation of polyunsaturated fatty acids (*p* = 0.001 and *p* = 0.015, respectively), while the sham operation did not affect the levels of NOX2 or 4-HNE compared with the control conditions (*p* = 0.519 and *p* = 0.932, respectively, Fig. 3).

### AMPK/Akt/eNOS signaling pathway proteins expression in renal arties with and without RDN

**Fig. 4:**
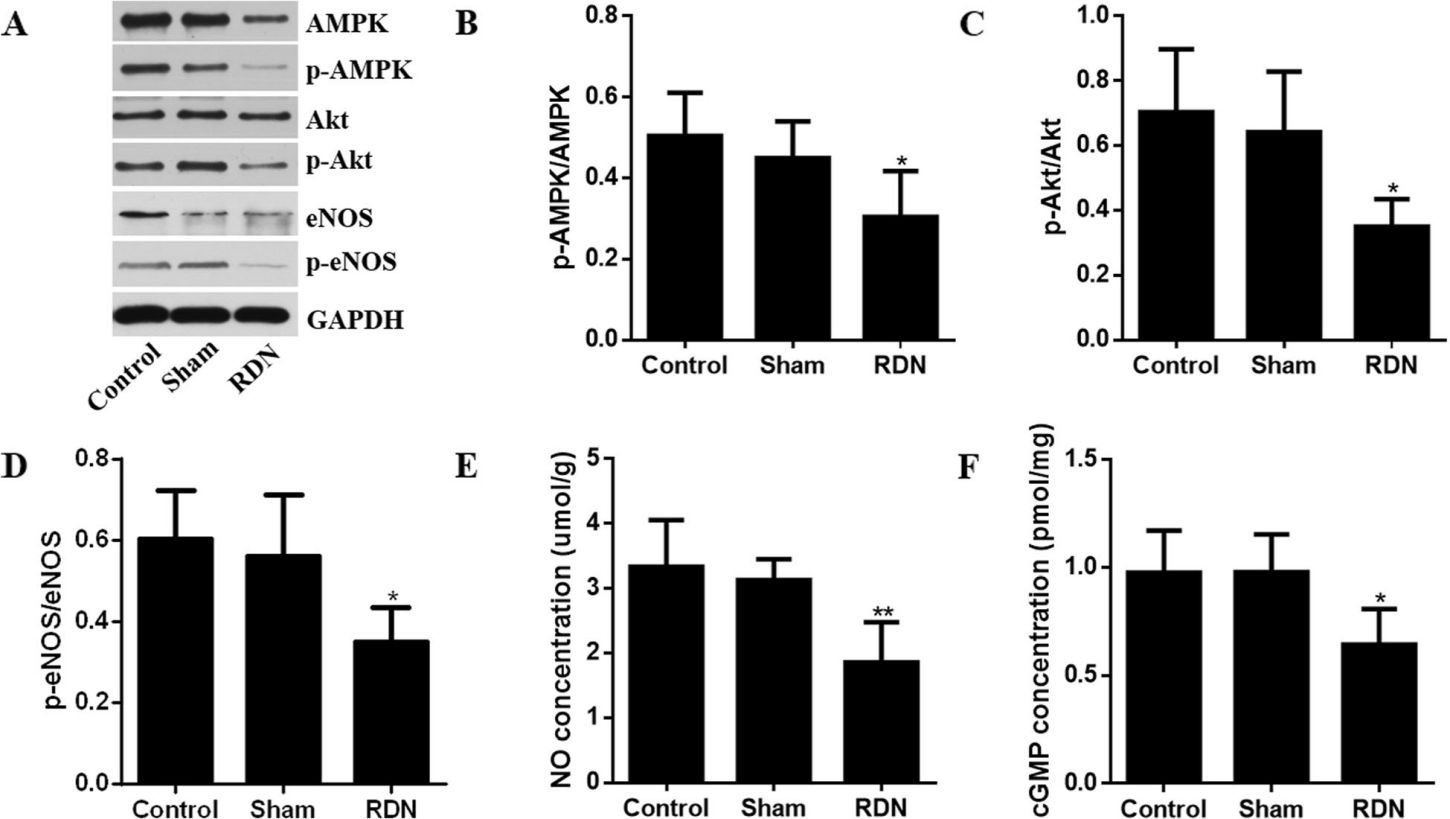
Effects of RDN on the signaling pathway of NO production. Representative images of Western blots of AMPK/Akt/eNOS pathway protein expression (**a**) and the corresponding quantitation (**b**-**d**); NO (**e**) and cGMP levels (**f**) in pig renal arteries. Data are expressed as the mean ± standard deviation. *p < 0.05, **p < 0.01 vs. sham group; *n* = 5 per group. Abbreviations: RDN, renal denervation; NO, nitric oxide; cGMP, cyclic guanosine monophosphate; AMPK, adenosine 5′-monophosphate (AMP)-activated protein kinase; eNOS, endothelial nitric oxide synthase; Akt, protein kinase B; GAPDH, glyceraldehyde phosphate dehydrogenase

As shown in Fig. 4, compared to the sham treatment, RDN significantly suppressed the phosphorylation levels of AMPK, Akt and eNOS (*p* = 0.046, p = 0.015 and *p* = 0.018, respectively) and significantly reduced the levels of NO and cGMP (*p* = 0.004 and p = 0.015, respectively) in obese pigs. However, no significant differences were observed between the control and sham groups (*p* = 0.427, *p* = 0.557, *p* = 0.594, *p* = 0.191, and *p* = 0.467, respectively).

### Expression of endothelin system proteins in renal arties with and without RDN

**Fig. 5:**
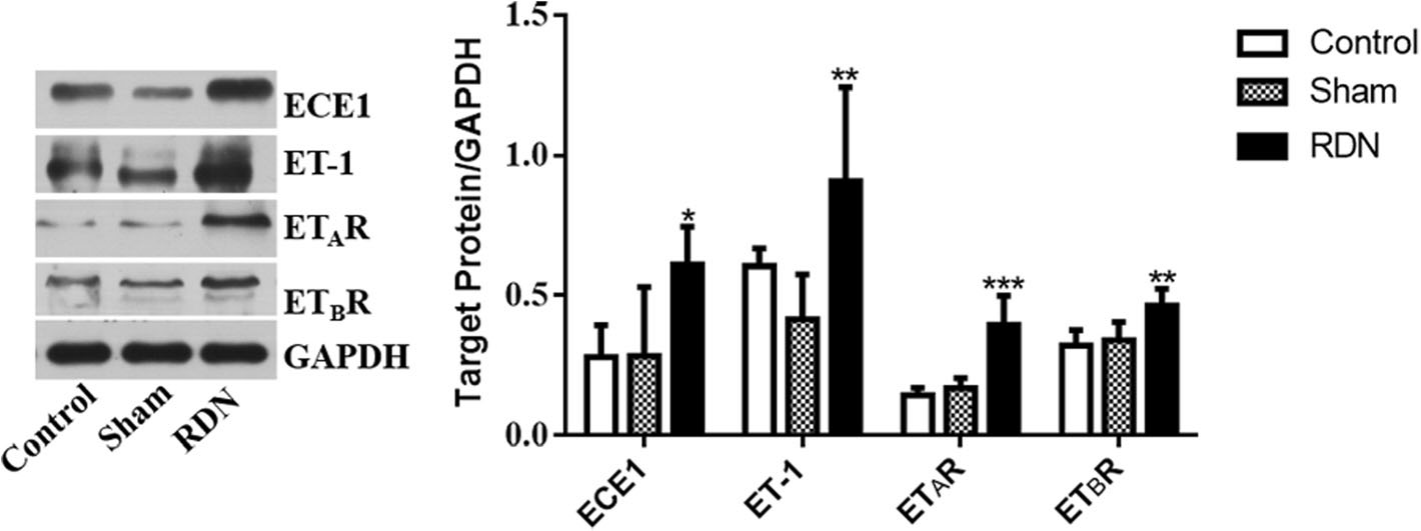
Representative images of Western blots of ECE1, ET-1, ET_A_R and ET_B_R protein expression (left panel) and the corresponding quantitation (right panel) in the control, sham and RDN groups. Data are expressed as the mean ± standard deviation. *p < 0.05, **p < 0.01, ***p < 0.001 vs. sham group; *n* = 5 per group. Abbreviations: RDN, renal denervation; ET-1, endothelin-1; ET_A_R, endothelin A receptor; ET_B_R, endothelin B receptor; ECE 1, endothelin converting enzyme 1; GAPDH, glyceraldehyde phosphate dehydrogenase

The expression levels of ECE1, ET-1, ET_A_R and ET_B_R were determined (Fig. 5) and were similar between the control and sham groups (*p* = 0.972, p = 0.191, *p* = 0.540 and *p* = 0.648, respectively). Compared to the sham group, the RDN group had significantly increased expression levels of ECE1, ET-1, ET_A_R and ET_B_R (*p* = 0.012, *p* = 0.004, *p* = 0.000 and *p* = 0.007, respectively).

### NF-κB activation of renal arteries with RDN

**Fig. 6:**
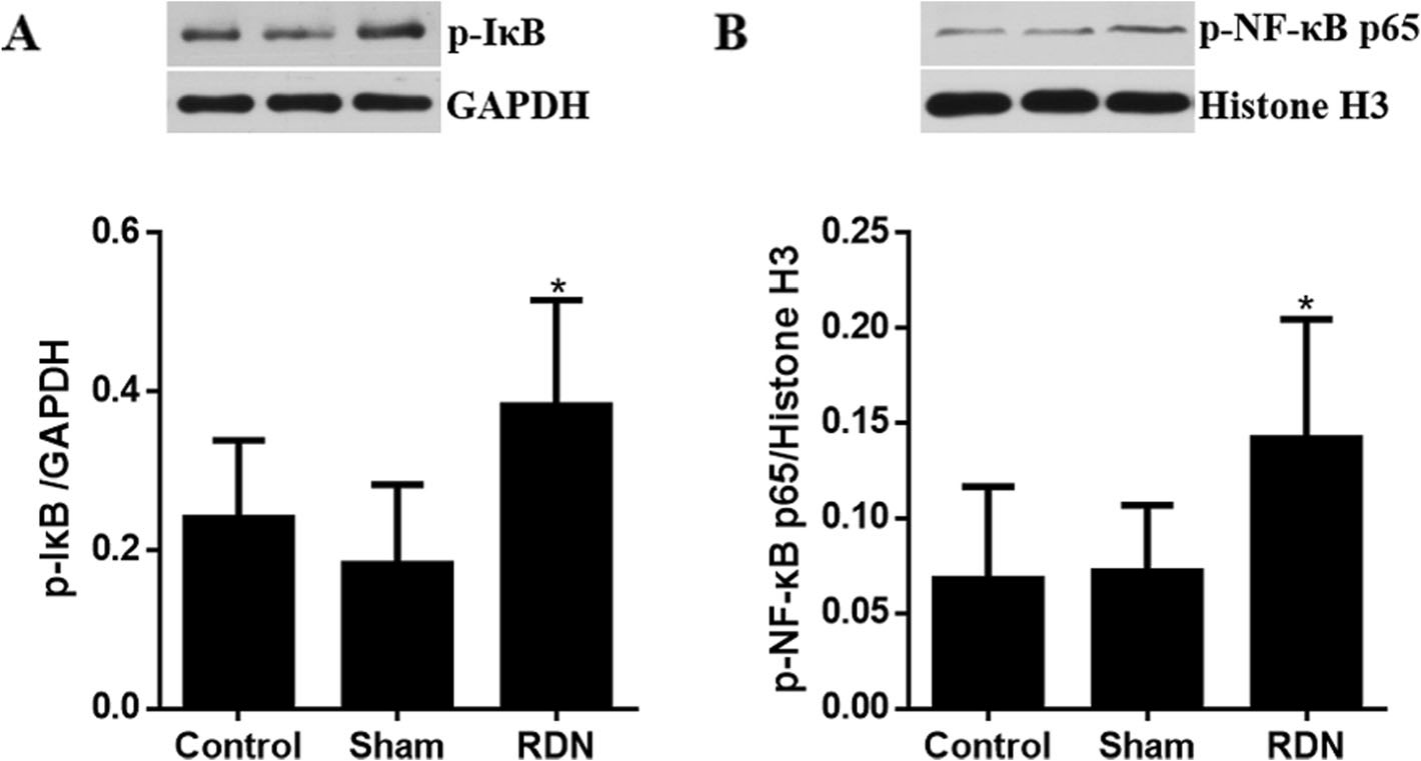
Effects of RDN on the levels of cytoplasmic p-IκB and nuclear p-NF-κB p65 in renal arteries. Levels of (**a**) p-IκB and (**b**) p-NF-κB p65 were determined by Western blot analysis. Data are expressed as the mean ± standard deviation. *p < 0.05 vs. sham group, *n* = 5 per group. Abbreviations: RDN, renal denervation; GAPDH, glyceraldehyde phosphate dehydrogenase; p-IκB, phosphorylated-I kappa B; p-NF-κB p65, NF-kappa B p65

As presented in Fig. 6, RDN significantly enhanced the cytoplasmic expression of p-IκB and the nuclear expression of p-NF-κB p65 compared with the sham treatment (*p* = 0.037 and *p* = 0.039, respectively). However, no significant differences in the activation of cytoplasmic p-IκB and nuclear p-NF-κB p65 were observed between the control and sham groups (*p* = 0.809 and *p* = 0.899, respectively).

### Effects of RDN on the expression of ET_A_R and ET_B_R

**Fig. 7:**
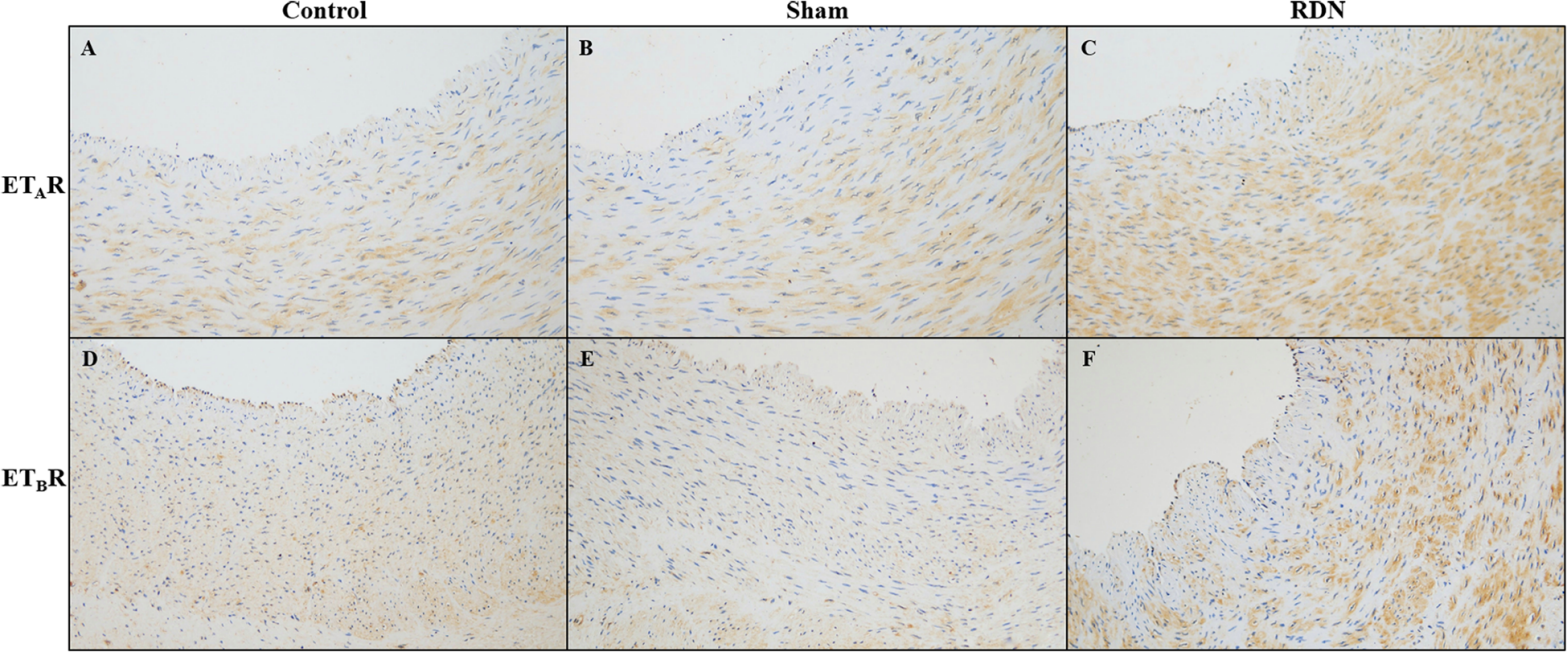
Representative images of the immunohistochemical detection of target protein expression in renal arteries (× 200). ET_A_R in the control (**a**), sham (**b**) and RDN (**c**) groups; ET_B_R in the control (**d**), sham (**e**) and RDN (**f**) groups. Abbreviations: RDN, renal denervation; ET_A_R, endothelin A receptor; ET_B_R, endothelin B receptor

Immunohistochemistry was used to compare the expression of ET_A_R and ET_B_R among the 3 studied groups (Fig. 7). RDN significantly upregulated the expression levels of ET_A_R in VSMCs compared to the sham operation. The expression of ET_B_R, which is mainly expressed in endothelial cells under normal physiological conditions [12], was also significantly increased in VSMCs after RDN treatment. However, the expression levels and staining intensity of ET_A_R and ET_B_R in the control and sham groups were consistent. Additionally, the expression and staining intensity of ET_A_R were significantly higher than those of ET_B_R in the VSMCs of the control and sham groups, while the expression and staining intensity of these two receptors in VSMCs were similar within the RDN group.

## Discussion

The major findings of the current study were as follows: (1) RDN aggravated endothelial endocrine dysfunction and intimal thickening of renal arteries; (2) RDN significantly increased the renal arterial level of oxidative stress; and (3) RDN significantly activated the nuclear translocation of NF-κB and increased the risk of atherosclerosis of renal arteries.

The endothelium regulates vascular wall homeostasis by releasing vasodilators such as NO and vasoconstrictors such as ET-1. NO is synthesized through the enzymatic conversion of the amino acid L-arginine by eNOS, which can be phosphorylated by an AMPK-dependent pathway at Ser1177 and the PI3K/Akt signaling pathway [13]. In this study, RDN significantly downregulated the phosphorylation level of Akt at position Ser473 and AMPKα at position Thr172 compared to the sham operation. Thus, RDN significantly suppressed the activation of eNOS, decreased the production and activity of NO and cGMP, and can eventually lead to weakened vasodilation, which is one of the earliest events in the pathogenesis of atherosclerosis [14].

ET-1 is produced by ECE1 and regulates vascular tone via ET_A_R and ET_B_R. ET_A_R is located on VSMCs and contributes to their vasoconstricting properties. ET_B_R is distributed in endothelial cells, in which it promotes vasodilation by releasing NO, and in smooth muscle cells, where it mediates vasoconstriction. Accumulating evidence suggests that ET-1 expression is upregulated in atherogenesis, which induces endothelial dysfunction, VSMC proliferation and migration and vessel constriction [15]. A previous study indicated that there are higher levels of ET_A_R and ET_B_R in VSMCs in the medial region of experimental atherosclerotic lesions than in VSMCs of normal arteries. Mixed ET_A_R and ET_B_R receptor antagonism can decrease intimal thickening and reduce atherosclerosis caused by the inflammatory response [16]. Consistent with the above evidence, we found that protein expression of ECE1, ET-1 and its receptors significantly increased in the ablated arteries of the RDN group compared with the arteries of the sham group. Moreover, immunohistochemical results showed a stronger immunostaining intensity of ET_A_R and an increase in ET_B_R immunoreactivity in renal arterial VSMCs in the RDN group compared with those in the sham group, which suggested an increasing risk of atherosclerosis after RDN. In normal arterial smooth muscle cells, the expression of ET_A_R is significantly greater than that of ET_B_R, but in atherosclerotic vessels, the levels of the two molecules were similar [17], which was demonstrated in our findings. The changes in the relative levels of endothelin receptor subtypes may be due to the switching of ET_A_R expressed predominantly in contractile phenotype VSMCs to ET_B_R expressed preferentially in synthetic phenotype VSMCs in the process of atherosclerosis [17].

Endothelial dysfunction is clearly involved with oxidative stress [18]. NADPH oxidase is a superoxide-synthesizing enzyme and is detected by enhanced expression of the NADPH oxidase subunit NOX2 in atherosclerotic arteries [18]. Furthermore, 4-HNE is the one of the most abundant and cytotoxic products of lipid peroxidation of polyunsaturated fatty acids, and is regarded as an important marker of oxidative stress and increased in atherosclerosis [19]. In this study, the western blotting results suggested that NOX2 and 4-HNE expression were obviously upregulated in the RDN group compared with the sham group, which suggested aggravated endothelial dysfunction in HFD-fed pigs treated with RDN. Additionally, NF-κB is an important transcription factor that activates inflammatory responses and contributes to early events in the development of atherosclerosis [20]. NF-κB (a heterodimer with subunits p50 and p65) binds to the inhibitor protein IκB in the cytosol in an inactive state, and NF-κB is activated and translocated freely into the nucleus after IκB phosphorylation and degradation in a pathological state [21]. In the present study, increased NF-κB activation, as indicated by significantly upregulated expression of cytoplasmic p-IκB and nuclear p-NF-κB p65, was observed in pigs following RDN compared to the sham operation. Experimental studies suggested that an ET_A_R antagonist can block the expression of the kinin B_1_ receptor associated with oxidative stress and inhibit NF-κB activation [22]. Therefore, upregulated ET-1 levels after RDN may contribute to increased activation of NF-κB and oxidative stress.

Our study suggested that body weight and TC significantly increased in all pigs after a HFD for 6 months. Moreover, HFD also elevated BP in control and sham groups pigs. These were consistent with the reports that HFD-induced obesity can lead to abnormal lipid metabolism disorders and endothelial dysfunction, which can promote the occurrence and the development of hypertension [23, 24]. Renal afferent sympathetic nerve fibers transmit signals to central nervous system which generates and sends sympathetic signals to various targets including heart and kidney, resulting in activating or depressing their sympathetic nervous activity and the changes of BP. Renal efferent sympathetic nerve fibers regulate BP by affecting the activity of renin-angiotensin-aldosterone system (RAAS), renal hemodynamics and renal sodium and water excretion [25, 26]. Overactive renal afferent and efferent sympathetic nervous can activate RAAS, promote sodium and water retention, decrease renal blood flow and eventually elevate BP [25, 26]. Our data indicated that eliminating the overactivated renal afferent and efferent sympathetic nervous in renal arteries by RDN significantly reduced SBP and DBP when compared with sham group. Some studies have shown that radiofrequency ablation energy targeting removing sympathetic nervous applied to the arterial wall induced transmural tissue coagulation and loss of endothelium in an acute phase, and transmural media damage coexisted with the presence of proteoglycan at 6 months after RDN [6, 7, 27]. Vascular endothelial cell injury can cause abnormal proliferation and migration, induce the change from a contractile phenotype to a synthetic phenotype of VSMCs, cause vascular wall thickening, and eventually lead to hypertension and atherosclerosis, which is a lipid-initiated, progressive, inflammatory intimal disease [28]. These findings support the present data indicating that because of the dual stimulation of pathophysiological factors and endothelial mechanical injury, the intima was thicker in HFD-fed pigs treated with RDN than in pigs treated with a sham operation. Intimal thickening is associated with early atherosclerosis [29]. A published case report described a patient with resistant hypertension whose renal arteriography findings were normal before RDN (170/90 mmHg) and whose BP was effectively controlled for 3 months after RDN (140/70 mmHg), but the patient developed 75% renal artery stenosis near the ablation site, hypertension recurrence 6 months after RDN (180/92 mmHg), and a decrease in systolic BP to 150 mmHg 1 month after stent implantation [30]. The degree of rapid progression of renal arterial stenosis induced by RDN is unclear, and intimal thickening relevant to RDN may play an essential role [29]. In the present experimental results, no significant difference was observed between the control and sham groups, which suggested the safety of renal arteriography.

## Conclusions

This study provided evidence that RDN can aggravate renal arterial endothelial endocrine dysfunction and intimal thickening, and increase the risk of atherosclerosis in HFD-fed pigs. Thus, in clinical practice, we should monitor endothelial function in the ablated renal arteries and serum lipids after RDN, and provide prophylactic anti-inflammatory, anti-endothelial dysfunction and lipid-lowering treatments if necessary, to prevent complications.

## Limitations

The experimental pig was not a hypertensive pig model, and cannot fully reflect the effects of hypertension on endothelial function, and the changes of endothelial function after RDN. In addition, this study mainly discussed the effects of RDN on the risks of renal arterial atherosclerosis from the perspective of pathology and molecular biology. There was a lack of detection using imaging techniques such as Intravascular Ultrasound (IVUS) and Optical Coherence Tomography (OCT) which allow high-resolution imaging and help us more clearly observed vascular changes after RDN and more comprehensively explain the effects of RDN on renal arteries.

## Data Availability

The datasets that support the findings of this study are available from the corresponding author on reasonable request.

## Abbreviations

TC: Total cholesterol
TG: Triglyceride
HDL-C: High density lipoprotein cholesterol
LDL-C: Low density lipoprotein cholesterol
Scr: Serum creatinine
SBP: Systolic blood pressure
DBP: Diastolic blood pressure
RAAS: Renin-angiotensin-aldosterone system
RDN: Renal denervation
NO: Nitric oxide;ET-1:endothelin-1
HFD: High-fat diet
cGMP: Cyclic guanosine monophosphate
NOX2: NADPH oxidase 2
4-HNE: 4-hydroxynonenal;ET-1:endothelin 1
BP: Blood pressure
ET_A_R: Endothelin a receptor
ET_B_R: Endothelin B receptor
ECE 1: Endothelin converting enzyme 1
AMPK: Adenosine 5′-monophosphate (AMP)-activated protein kinase
eNOS: Endothelial nitric oxide synthase
Akt: Protein kinase B
GAPDH: Glyceraldehyde phosphate dehydrogenase
p-IκB: Phosphorylated-I kappa B
p-NF-κB p65: NF-kappa B p65
HE: Hematoxylin and eosin
BSA: Bovine serum albumin
DAB: Diaminobenzidine
VSMCs: Vascular smooth muscle cells
